# Combination of Repurposed Drug Diosmin with Amoxicillin-Clavulanic acid Causes Synergistic Inhibition of Mycobacterial Growth

**DOI:** 10.1038/s41598-019-43201-x

**Published:** 2019-05-01

**Authors:** Anju Choorakottayil Pushkaran, Vivek Vinod, Muralidharan Vanuopadath, Sudarslal Sadasivan Nair, Shantikumar V. Nair, Anil Kumar Vasudevan, Raja Biswas, Chethampadi Gopi Mohan

**Affiliations:** 1 Center for Nanosciences and Molecular Medicine, Amrita Institute of Medical Sciences and Research Centre, Amrita Vishwa Vidyapeetham, Ponekkara, Kochi, 682 041 Kerala India; 2 Department of Microbiology, Amrita Institute of Medical Sciences and Research Centre, Amrita Vishwa Vidyapeetham, Ponekkara, Kochi, 682 041 Kerala India; 3 School of Biotechnology, Amrita Vishwa Vidyapeetham, Clappana, Kollam, 690 525 Kerala India

**Keywords:** Antibiotics, Virtual drug screening

## Abstract

Effective therapeutic regimens for the treatment of tuberculosis (TB) are limited. They are comprised of multiple drugs that inhibit the essential cellular pathways in *Mycobacterium tuberculosis* (*Mtb*). The present study investigates an approach which enables a combination of Amoxicillin-Clavulanic acid (AMC) and a repurposed drug for its synergistic effect towards TB treatment. We identified Diosmin (DIO), by targeting the active site residues of L,D-transpeptidase (Ldt) enzymes involved in *Mtb* cell wall biosynthesis by using a structure-based drug design method. DIO is rapidly converted into aglycone form Diosmetin (DMT) after oral administration. Binding of DIO or DMT towards Ldt enzymes was studied using molecular docking and bioassay techniques. Combination of DIO (or DMT) and AMC exhibited higher mycobactericidal activity against *Mycobacterium marinum* as compared to individual drugs. Scanning electron microscopy study of *M. marinum* treated with AMC-DIO and AMC-DMT showed marked cellular leakage. *M. marinum* infected *Drosophila melanogaster* fly model showed an increased fly survival of ~60% upon treatment with a combination of AMC and DIO (or DMT). Finally, the enhanced *in vitro* antimicrobial activity of AMC-DIO was validated against *Mtb* H37Ra and a MDR clinical isolate. Our results demonstrate the potential for AMC and DIO (or DMT) as a synergistic combination for the treatment of TB.

## Introduction

*Mycobacterium tuberculosis (Mtb)* is one of the leading human pathogens and causative agent of tuberculosis (TB). According to the World Health Organization (WHO) annual report, one fourth of the world’s population is infected with TB^1^. A standard treatment regimen for TB involves a combination therapy of Rifampicin (RIF), Isoniazid (INH), Pyrazinamide and Ethambutol for the first 2 months, followed by RIF and INH for an additional 4 months. In recent years, 0.49 million people suffered annually from multi-drug resistant (MDR)-TB with a cure rate of only 52%^1^. In MDR-TB, the bacilli are resistant to both RIF and INH and the combination chemotherapy lasts for two years^2^. Further, in extensively drug resistant (XDR)-TB, *Mtb* is resistant to RIF, INH,any of the fluoroquinolones (eg., Levofloxacin, Moxifloxacin), and one among the three second-line injectable drugs (Amikacin, Capreomycin or Kanamycin)^3^. It was reported that 6.2% of the MDR-TB cases are XDR-TB^1^. A rapid increase in the emergence of MDR and XDR-TB underlines the urgent need for the development of new drugs with novel mechanisms of action.

Enzymes involved in the *Mtb* cell wall biosynthetic pathway are absent in eukaryotic hosts, and therefore are attractive targets for TB drug development. The peptidoglycan (PG) of the *Mtb* cell wall is comprised of repeating disaccharide sugar units of *N*-glycolylmuramic acid (MurNGlyc) linked to *N*-acetylglucosamine. MurNGlyc unit is lactyl-linked to a linear stem peptide consisting of amino acids L-Alanine, D-Glutamic acid, *meso*-diaminopimelic acid (*meso*-DAP), D-Alanine (D-Ala) and D-Ala^4,5^. Two different types of PG cross-linkages (classical and non-classical) are observed in *Mtb*. In the classical type of PG cross-linkage, the carboxyl group of D-Ala^4^ is linked to *meso*-DAP^3^ of the neighbouring pentapeptide (D-Ala^4^→*meso*-DAP^3^)^6^. This reaction is catalysed by the enzyme D,D-transpeptidase. *β*-lactam antibiotics (eg., Amoxicillin (AMX)) target this key enzyme^7^. *Bacillus subtilis*, *Enterococcus faecium, Mycobacterium* species and related species contain non-classical type PG cross-linkage formed between the neighbouring *meso*-DAP^3^ residues, which are catalysed by the L,D-transpeptidase (Ldt) enzymes leading to *β*-lactam resistance^6,8–11^. In *Mtb*, mainly two functional Ldt paralogs, Ldt_Mt1_ and Ldt_Mt2_ are present and Ldt_Mt2_ is more predominantly expressed than Ldt_Mt1_^8,10^. Loss of Ldt_Mt1_ and Ldt_Mt2_ alters cellular morphology, size, virulence and *β*-lactam resistance^8,12^. Hence, *Mtb* Ldt enzymes represent an important druggable target for the treatment of TB^13^.

Emerging evidence suggest that the carbapenem subclass of *β*-lactam antibiotics, such as Meropenem (MEM), Imipenem, and Ertapenem, inhibit both the D,D-transpeptidase and Ldt enzymes^13–15^. *In vitro* and *in vivo* studies recently demonstrated the susceptibility of *Mtb* to carbapenems^13,15–17^. Hugonnet *et al*., reported that a combination of MEM and Clavulanic acid kills the persisting and drug resistant tubercle bacilli^18^. The molecular mechanism of Ldt enzyme inhibition by MEM and other carbapenems are well studied^19–23^. However, the major drawback of MEM is its low oral bioavailability and parenteral administration to the patients^24^.

Recently, many drugs have been repurposed for treating various bacterial infections, including TB. Drug repurposing/repositioning involves identifying novel druggable targets for the existing drugs and this includes Linezolid, Simvastatin, Metformin, Colesevelam, Zidovudine^25,26^. This technique significantly reduces the cost and time involved in the traditional drug discovery process^27,28^. The main objective of the present study involves repurposing of FDA approved drugs for TB combination treatment, by integrating *in silico, in vitro* and *in vivo* techniques. We computationally evaluated repurposing of an orally bioavailable FDA approved drug, Diosmin (DIO) using structure-based drug design method. Further, a synergistic *in vitro* and *in vivo* anti-mycobacterial effect was observed when DIO was used in combination with Amoxicillin-Clavulanic acid (AMC) against live bacilli. The efficacy of AMC-DIO combination against *Mtb* and an MDR-*Mtb* clinical isolate was also assessed.

## Results and Discussion

Emerging antibiotic resistance is major concern in the current TB chemotherapy. The inhibition of both D,D-transpeptidase and Ldt enzymes prevents the formation of both classical and non-classical PG cross-linking, which is involved in the *Mtb* growth, survival and drug resistance. We repurposed an FDA approved drug (DIO) against key *Mtb* druggable targets Ldt_Mt1_ & Ldt_Mt2_ by using a structure-based drug design method. The anti-mycobacterial activity of the repurposed drug (DIO) was further validated by use of *in vitro* and *in vivo* techniques.

### Multiple Sequence Alignment studies of the Ldt enzymes of *Mtb*, *M. marinum* and *M. smegmatis*

**Figure 1:**
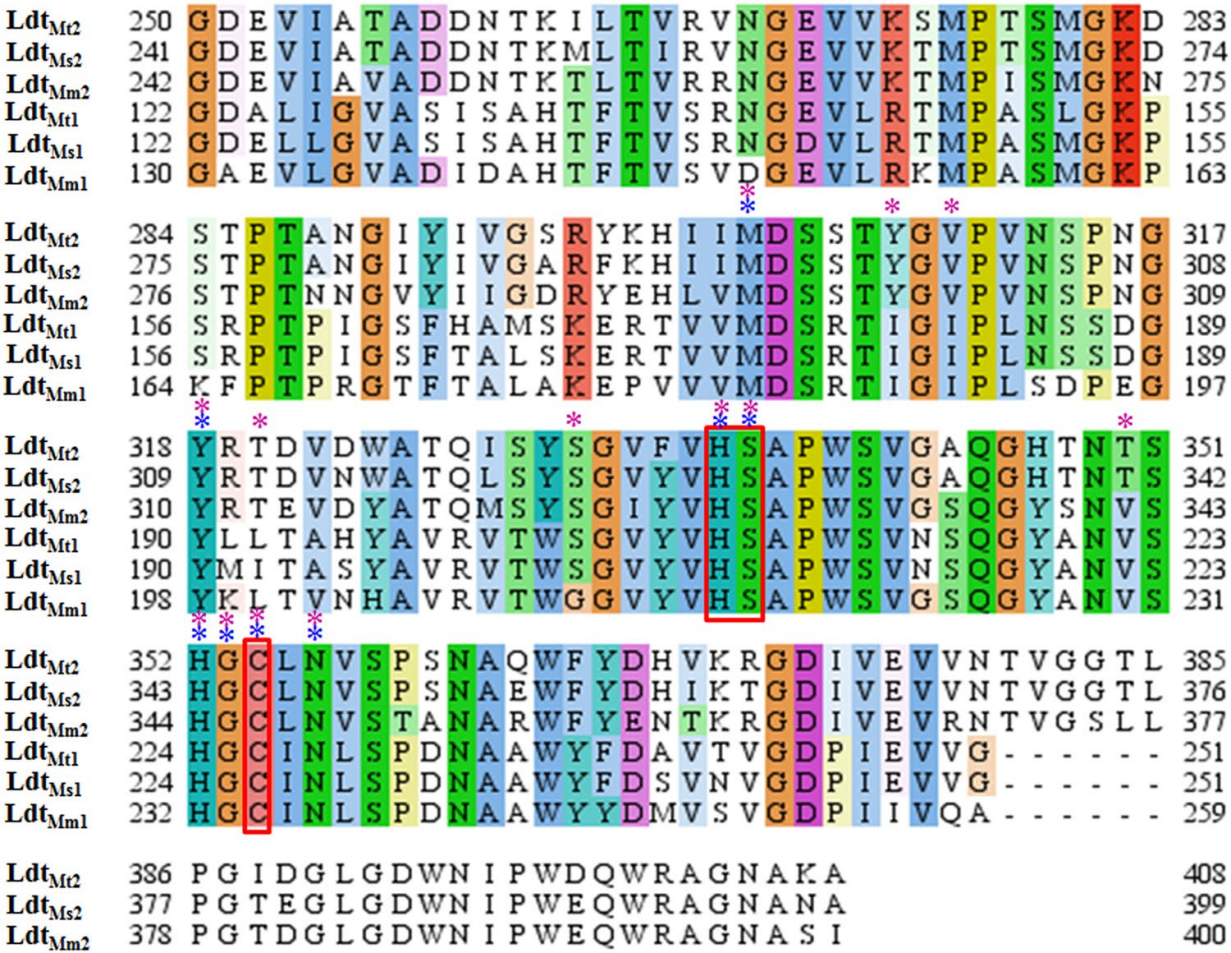
The multiple sequence alignment of L,D transpeptidases of different mycobacterial species. The sequence alignment of the catalytic region of L,D transpeptidases of *Mtb* (Ldt_Mt1_ & Ldt_Mt2_), *M. marinum* (Ldt_Mm1_ & Ldt_Mm2_) and *M. smegmatis* (Ldt_Ms1_ & Ldt_Ms2_). The red boxes represent the three important catalytic residues Cys, His and Ser. The blue and pink asterisks indicate the reported key residues^9,14,19,22,23^ involved in the inhibitor binding of Ldt_Mt1_ and Ldt_Mt2_ respectively.

Initially, we performed multiple sequence alignment (MSA) of Ldts of *Mtb*, *Mycobacterium marinum* (Ldt_Mm1_and Ldt_Mm2_), and *Mycobacterium smegmatis* (Ldt_Ms1_ and Ldt_Ms2_), in order to understand the evolutionary sequence conservation among these different mycobacterial species. *Mtb* Ldt_Mt1_ shares a sequence identity of 81.7% and 56.9% towards Ldt_Mm1_ and Ldt_Ms1,_ and *Mtb* Ldt_Mt2_ shares a sequence identity of 82.2% and 67.3% towards Ldt_Mm2_ and Ldt_Ms1_ respectively, as shown in Fig. 1. The primary sequence analysis suggests that both the Ldt enzymes of *Mtb*, *M. marinum* and *M. smegmatis* mycobacterial species share high sequence identity, suggesting its similar three dimensional (3D) structural fold.

Recent studies showed that the carbapenems such as MEM, ETP, and Tebipenem, bind to the key catalytic triad residues Cys226, His208, Ser209 of Ldt_Mt1_ and Cys354, His336, Ser337 of Ldt_Mt2_^14,19–22^. X-ray crystallographic and kinetic studies revealed that Met175/303, Tyr190/318 His224/352, Gly225/353, and Asn228/356 residues of Ldt_Mt1_/Ldt_Mt2_ form key non-covalent interactions with the inhibitor^9,14,19,22,23^. Further, these residues are well conserved in the MSA analysis, as shown in Fig. 1. Thus, the probability of a single compound to bind with the key catalytic residues Cys, His and Ser of both Ldt_Mt1_ and Ldt_Mt2_ enzymes were strongly predicted by MSA studies.

### Drug repurposing by structure-based virtual screening against Ldt_Mt1_ and Ldt_Mt2_

**Figure 2:**
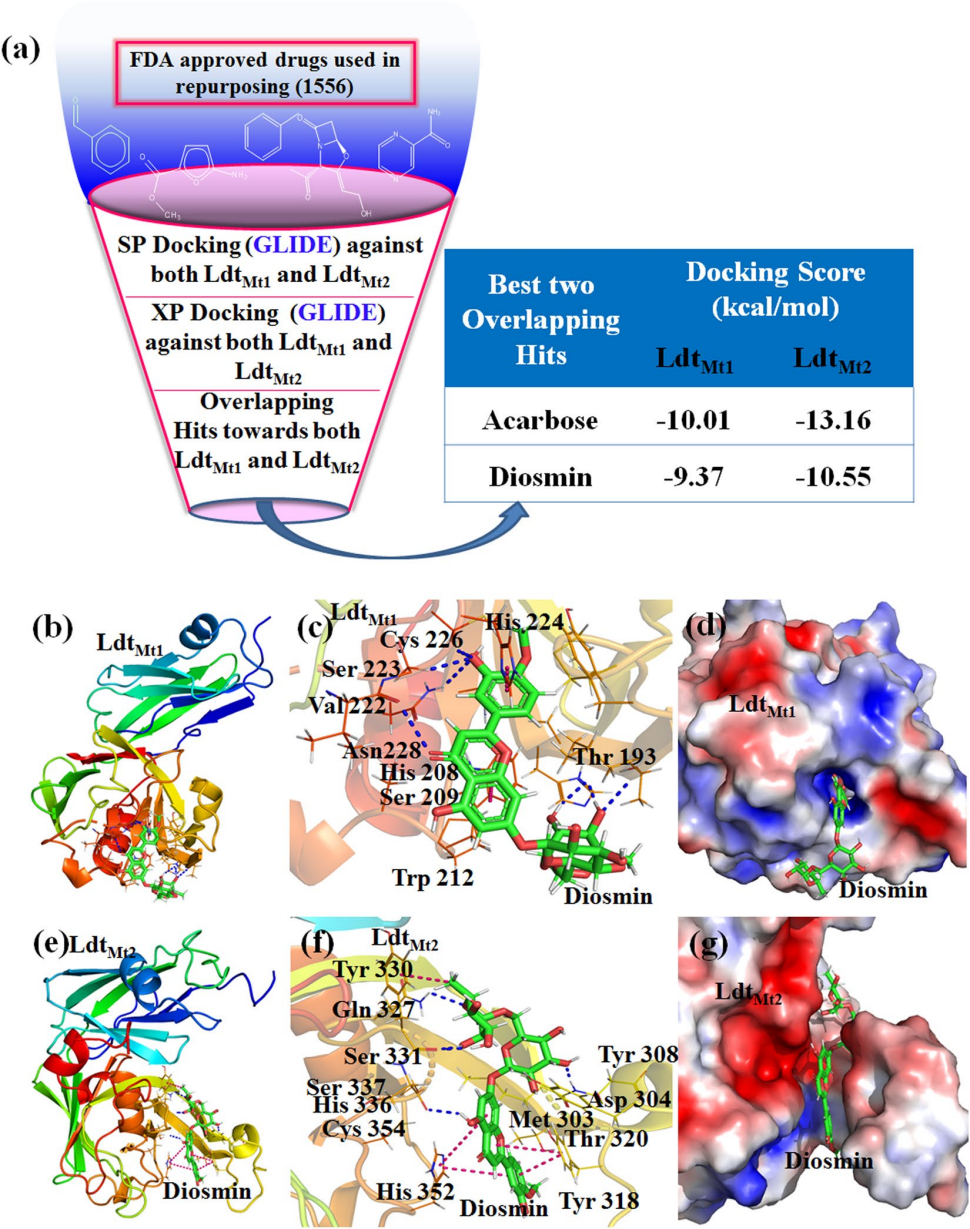
Drug repurposing by structure-based virtual screening against Ldt_Mt1_ and Ldt_Mt2_. (**a**) Virtual screening workflow adopted for the drug repurposing study with the highest ranked overlapping hits towards both Ldt_Mt1_ and Ldt_Mt2_ enzymes. Molecular interactions analysis of the Ldt-Diosmin complexes: (**b**) Docked complex of Ldt_Mt1_ with Diosmin (DIO); (**c**) molecular interactions of DIO towards the active sites of Ldt_Mt1_ and (**d**) Molecular electrostatic potential (MEP) surface map of the Ldt_Mt1_ - DIO active site regions. (**e**) Docked complex of Ldt_Mt2_ with DIO (**f**) molecular interactions of DIO towards the active sites of Ldt_Mt2_and (**g**) MEP surface map of the Ldt_Mt2_ - DIO active site regions. The proteins are shown as cartoon and DIO is represented as green sticks. The interacting amino acid residues at the active site regions are shown as lines and the hydrogen bonding and hydrophobic interactions are shown as blue and pink dotted lines respectively.

Crystal structure of both *Mtb* Ldt enzymes reveal that the key catalytic triad residues Cys226, His208, Ser209 of Ldt_Mt1_and Cys354, His336, Ser337 of Ldt_Mt2_ are directly involved in the *Mtb* non-classical PG cross-linkage^19,22^. We selected Ldt_Mt1_ & Ldt_Mt2_ molecular targets for our computer-aided drug repurposing study. The FDA approved drugs (1556) from the DrugBank database were used for the high-throughput virtual screening (VS) by molecular docking technique against the active sites of Ldt_Mt1_ and Ldt_Mt2_ enzymes. Best 10% of the Glide high scoring drugs common to both VS hits against Ldt_Mt1_ and Ldt_Mt2_ were selected, which include Acarbose and DIO (Fig. 2a and Table 1**)**. Acarbose, was obtained as the best VS hit with a high docking score of −10.01 and −13.16 kcal/mol against Ldt_Mt1_ and Ldt_Mt2_ respectively, as shown in Table 1. Acarbose is used for the treatment of Type 2 diabetes mellitus and has a very low bioavailability. An earlier report suggested that Acarbose binds to trehalose synthase enzyme of *M. smegmatis*^29^. DIO was the second highest ranked VS hit with a docking score of −9.37 kcal/mol for Ldt_Mt1_ and −10.55 kcal/mol for Ldt_Mt2_ enzymes, shown in Table 1. DIO is a bioflavonoid compound used to treat venous disorders and also prescribed as a dietary supplement. It also possesses anti-inflammatory and anti-oxidant activities^30–32^. Since Acarbose has very low bioavailability (~2%)^33^, we selected DIO for our *in vitro* and *in vivo* validation studies.

**Table 1:** The molecular interactions of the best two virtually screened hits towards both the Ldt_Mt1_ and Ldt_Mt2_ enzymes.

S. No.	Drug	FDA approved indication	Glide docking score (kcal/mol)		Interacting amino acid residues^a^	
			Ldt_Mt1_	Ldt_Mt2_	Ldt_Mt1_	Ldt_Mt2_
1	**Acarbose**	Type 2 Diabetes Mellitus	−10.01	−13.16	**Trp 212**, **Gly 189**, **Leu 191**, **His 224**, **Val 222**, **Asn 228**, **Thr 193**, **His 195**, **Ser 213**, **Val 222**	**Trp340**, **Asn356**, **His 352**, **Thr 350**, **Ser 331**, **Ser 351**, **Thr 320**, **Thr 350**, **Tyr 318**, His 352, Tyr 308, Met 303, Tyr 318
2	**Diosmin (DIO)**	Venous disease, Nutritional supplement	−9.37	−10.55	**His 224**, **Asn 228**, **Thr 193**, **Thr 194**, **Val 222**, **Ser 223**, His 208, Cys 226, Trp 212, His 224	**Asp 304**, **Gln 327**, **Tyr 330**, **Ser 331**, **Tyr 308**, **Thr 320**, His 352, Met303, Tyr 318

^a^Residues shown in bold take part in the hydrogen bonding interaction and others are involved in the hydrophobic interactions with the Ldt_Mt1_ and Ldt_Mt2_ enzymes. Experimentally reported amino acid residues involved in the PG cross-linking mechanisms are underlined (refs ^9,13,19,22,23^).

DIO is composed of a flavanoid moiety which is linked to a carbohydrate moiety via* O*-glycosidic bond (Supplementary Figure S1). Molecular docking studies revealed that DIO interacts with the Ldt_Mt1_ residues- His208, His224, Val222, Asn228, Thr193, Thr194, and Ser223 via hydrogen bonding, thiol group of Cys226 forms a π-bond with the flavonoid moiety of DIO; His224 and Trp213 are making π-π stacking interactions with DIO, as shown in Table 1 and Fig. 2b,c. Further, the interaction of DIO towards Ldt_Mt2_ showed that Asp304, Gln327, Tyr330, Ser331, Tyr308, and Thr320 residues are making hydrogen bonding interactions with DIO. Further, π-π stacking interactions were observed with Met303, His352, Tyr330 and Tyr318 residues, as shown in Table 1 and Fig. 2e,f. The interactions of DIO towards both the Ldt enzymes consisted of reported key residues His224, Cys226, Asn228 of Ldt_Mt1_ and Ser331, Tyr308, Thr320, His352, Met303, Tyr318 of Ldt_Mt2_^9,14,19,22,23^ required for the binding of carbapenem antibiotics, shown in Fig. 2 and Table 1. Thus, we observed that DIO adopts a similar binding mode as that of carbapenem with the active sites of both Ldt enzymes, using molecular docking studies.

It is well known that DIO is hydrolyzed into its aglycone form Diosmetin (DMT) by intestinal microflora enzymes^34,35^. Further, it was reported that DMT showed good synergistic inhibitory activity when used in combination with Erythromycin against pyruvate kinase of MRSA^36^. We analyzed the binding mode of DMT towards both the Ldt_Mt1_and Ldt_Mt2_ enzymes with respect to DIO for understanding its molecular mechanism of interactions. The GLIDE molecular docking and electrostatic potential surface mapping studies of these complexes showed that the flavonoid moiety of DIO is occupied in the active site tunnel of Ldts (Fig. 2d,g), and its carbohydrate moiety lies outside the active site tunnel. This implies that the flavonoid moiety of DIO is contributing towards the inhibition of both the Ldt enzymes. Further, the binding mode of DMT at the active sites of Ldt_Mt1_and Ldt_Mt2_ revealed a similar orientation and position as flavonoid moiety of DIO, with a Glide docking score of −6.48 kcal/mol and −6.23 kcal/mol respectively, as shown in Supplementary Fig. S2a–d.

To effectively block the PG biosynthesis of *Mtb*, simultaneous inhibition is essential for both the classical and non-classical PG cross linkages mediated by the D,D-transpeptidase and Ldt enzymes (Ldt_Mt1_and Ldt_Mt2_). The *β*-lactam antibiotic, AMX target D,D-transpeptidase enzyme. Glide molecular docking of AMX at the active site of D,D-transpeptidase (PDB ID:5CXW^37^) showed binding energy of −5.64 kcal/mol, and the molecular interactions of the complex (AMX-D,D transpeptidase) is shown in Supplementary Fig. S3a,b. These *in silico* results broadly reveal the mode of binding of DIO/DMT with *Mtb* Ldt enzymes as described above, and AMX with D,D-transpeptidase enzyme active sites, leading to a potential synergistic *Mtb* growth inhibition.

### Molecular dynamics simulation study of Ldt_Mt1_-DIO, Ldt_Mt2_-DIO and Ldt_Mt2_-MEM complex systems

**Figure 3:**
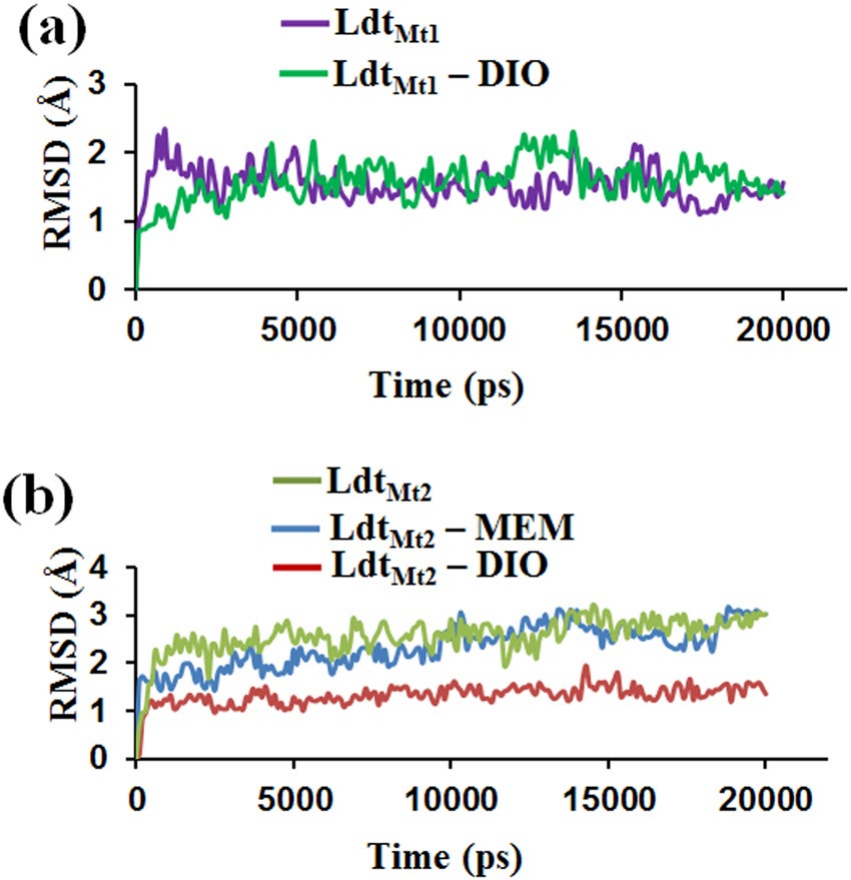
Molecular Dynamics (MD) simulation analysis of Ldt enzymes in complex with DIO. The root mean square deviation plot of (**a**) Ldt_Mt1_-DIO complex, (**b**) Ldt_Mt2_-DIO docked complex and Ldt_Mt2_-Meropenem (MEM) complex crystal structure during 20 ns MD simulation.

The 3D structural stability of Ldt enzymes in complex with DIO were analysed for 20 ns molecular dynamics (MD) simulations. Since, MEM is reported as a potent Ldt inhibitor^22^; Ldt_Mt2_-MEM crystal structure (PDB ID: 4GSU) was used as a control for our MD simulations. The 20 ns time interval of MD simulation trajectories were analysed to check the stability of the three different complexes, Ldt_Mt2_-MEM, Ldt_Mt1_-DIO, and Ldt_Mt2_-DIO. Good structural stability was observed for DIO in complex with Ldt_Mt1_ and Ldt_Mt2_ (Fig. 3a,b**)**. However, Ldt_Mt2_-MEM was found to be unstable throughout MD simulation, as indicated by the high backbone fluctuations with an average root mean square deviation (RMSD) of 2.34 Å (Fig. 3b). Whereas the average RMSD of Ldt_Mt1_-DIO and Ldt_Mt2_-DIO complexes were 1.5 Å and 1.3 Å respectively. Using MM-GBSA method, the binding free energy (ΔG) of Ldt_Mt2_-MEM complex was −15.10 kcal/mol and a two fold increase in ΔG was observed for the docked complexes of Ldt_Mt1_-DIO (−30.61 kcal/mol) and Ldt_Mt2_-DIO (−30.13 kcal/mol) (Supplementary Table S1). Our *in silico* results thus broadly indicate that DIO has a better binding affinity towards both the Ldt enzymes in comparison to that of MEM.

### Experimental binding studies of DIO (or DMT) and mycobacterial Ldt enzymes

**Figure 4:**
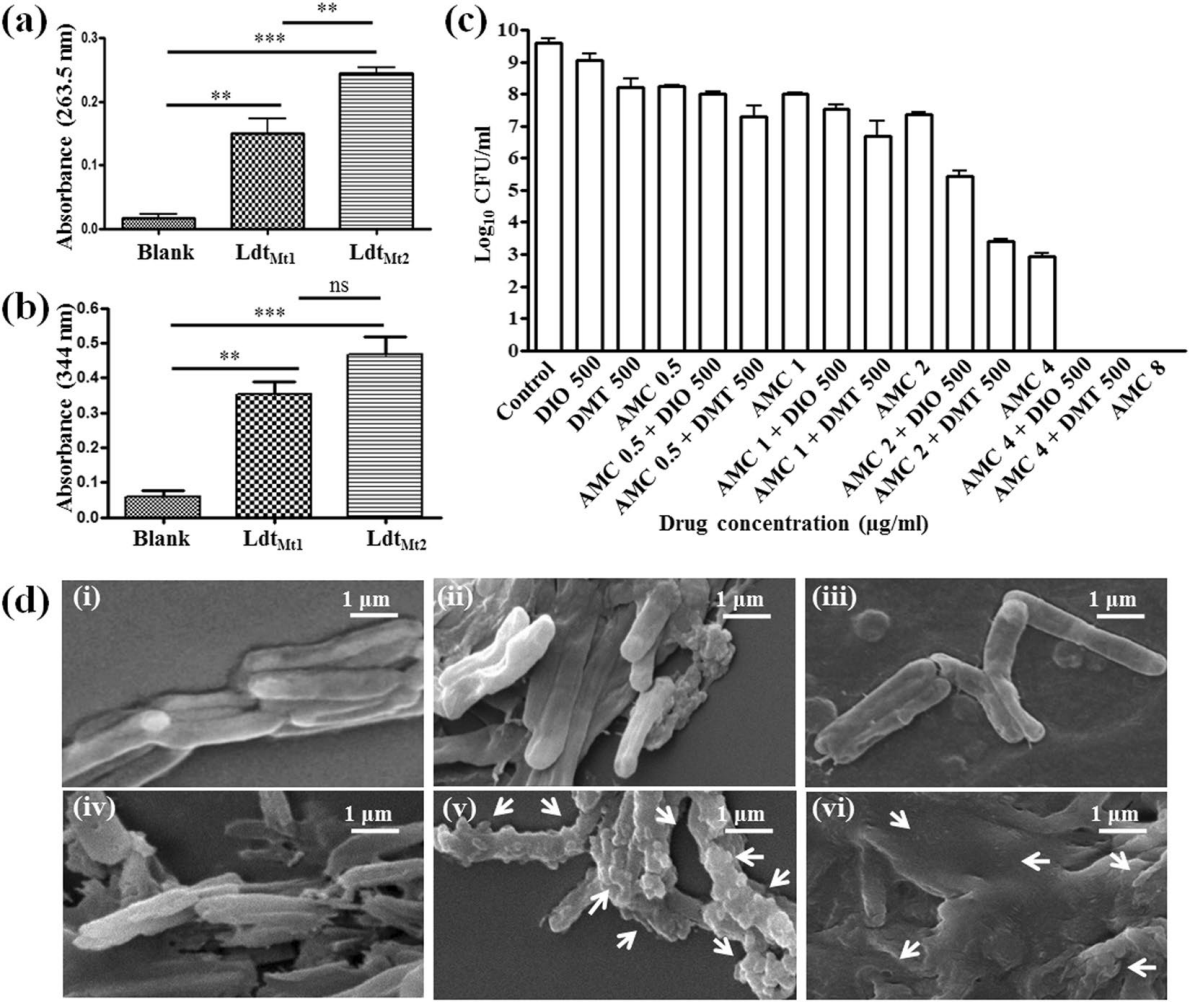
Experimental binding studies of DIO and Diosmetin (DMT) towards both the Ldts and the *in vitro* anti-mycobacterial activity of Amoxicillin-clavulanic acid (AMC)-DIO (or DMT) combination of drugs. Binding studies of (**a**) DIO and (**b**) DMT towards Ldt_Mt1_ and Ldt_Mt2_ indicated that both DIO and DMT have affinity towards both the proteins. (**c**) The comparative *in vitro* anti-mycobacterial activity of AMC, DIO, DMT, AMC-DIO and AMC-DMT (**d**) Scanning electron microscopic (SEM) images of *M. marinum* incubated (i) without any drugs with (ii) AMC (8 µg/ml), (iii) DIO (500 µg/ml), (iv) DMT(500 µg/ml), (v) AMC (8 µg/ml) - DIO (500 µg/ml) (vi) AMC (8 µg/ml) – DMT (500 µg/ml). The arrows in the SEM image represent the leakage of cellular components, large amorphous mass of cell debris and bulges in the cell surface. The statistical significance was analysed using one way ANOVA (***p < 0.001; **p < 0.01; ns, not significant).

The binding of Ldt_Mt1_ and Ldt_Mt2_ with DIO was studied using a bioassay method. The mycobacterial Ldt enzymes were allowed to bind to the Ni-NTA column and DIO (or DMT) was allowed to interact with the column bound Ldt enzymes. The complexes of Ldt-DIO (or Ldt-DMT) were eluted, and DIO (or DMT) was separated from the Ldt proteins by ultrafiltration. Moldovan *et al*. reported a sensitive spectrophotometric method for the determination of DIO with an absorbance maxima at 263.5 nm^38^. We also measured the optical density (OD) at 263.5 nm of the filtrate. DIO was found to be interacting with both the Ldt enzymes and the OD values of DIO in Ldt_Mt2_-DIO fractions was 0.245 and for Ldt_Mt1_-DIO fractions was 0.15, shown in Fig. 4a. This result suggests that DIO has stronger binding affinity towards Ldt_Mt2_ in comparison to that of Ldt_Mt1_ enzyme. Our molecular docking results also showed a similar trend of binding affinity when DIO was docked at the active site of Ldt_Mt2_ and Ldt_Mt1_, as described earlier (Table 1). Similarly, we analysed the binding of DMT towards both the mycobacterial Ldt enzymes. The absorption peak at 344 nm^39^ indicated the presence of DMT in the eluted fractions. The OD values of DMT in the eluted fraction of both Ldt_Mt1_ and Ldt_Mt2_ enzymes are almost similar, as shown in Fig. 4b. Thus, DMT has similar binding affinity towards both the enzymes. Hence, our experimental binding studies are in consonance with the computational molecular docking simulations, as described above.

### *In vitro* anti-mycobacterial activity studies of different drugs towards *M. marinum*

The *in-vitro* anti-mycobacterial activity of AMC, DIO, DMT alone and their combinations were tested against *M. marinum*, as described in the materials and methods section. No significant growth inhibition was observed when *M. marinum* was cultured in the presence of increasing concentrations of 100, 250 or 500 µg/ml DIO or DMT. Minimum bactericidal concentration (MBC) of AMC against *M. marinum* obtained in our study was 8 µg/ml. Next, increasing amount of AMC (0.5 µg/ml to 4 µg/ml) was added to the *M. marinum* cultures along with a fixed concentration of DIO or DMT (500 µg/ml), which showed a gradual decrease in the colony forming unit (CFU), as compared to AMC alone (Fig. 4c). The MBC of AMC was reduced to 4 µg/ml from 8 µg/ml concentration. Further, AMC-DMT combination of drugs exhibited higher anti-mycobacterial activity as compared to AMC-DIO, as indicated by the reduction in the CFU, as shown in Fig. 4c. Recently, Sridharan *et al*. predicted DIO to have a higher binding affinity towards the active site of mycobacterial panthothenate kinase by molecular docking studies^40^. However, this study also showed no anti-mycobacterial activity for DIO alone, and which is in good agreement with our present study. Further, the present results indicate that AMC has a synergistic anti-mycobacterial activity in combination with DIO or DMT against *M. marinum*.

### *M. marinum* treated with AMC-DIO (or DMT) combination of drugs severely altered its cell surface morphology

It is well known that PG layer of mycobacteria provides structural integrity and is essential for its growth and survival. Schoonmaker *et al*., demonstrated that the loss of Ldt_Mt1_ and Ldt_Mt2_ enzymes alter the cell surface morphology, shape and size of tubercle bacilli^12^. Based on these observations, we also hypothesised that the treatment of *M. marinum* with AMC-DIO or AMC-DMT combination of drugs would inhibit the PG cell wall cross-linking and cause changes in their cellular surface morphology. Scanning Electron Microscope (SEM) imaging study was performed for *M. marinum* treated with AMC, DIO or DMT individually and its combinations, AMC-DIO and AMC-DMT.

*M. marinum* with or without DIO (or DMT) drug treatment revealed a smooth cell surface, shown in Fig. 4d(i and iii). However, AMC treated *M. marinum* exhibited slightly corrugated cell surface with dents, as shown in Fig. 4d(ii). *M. marinum* cultured in the presence of DMT also exhibited slight changes in the cellular morphology (Fig. 4d(iv)). *M. marinum* on treatment with AMC-DIO or AMC-DMT showed dramatic changes in their cellular surface morphology consisting of unrecognizable large amorphous mass of cell debris (Fig. 4d(v and vi)). In addition, degraded cells appeared shrunk and corrugated with evident atrophy, and also bulges were present all over the mycobacterial cell surface. Also, *Mtb* cell wall has both DD-transpepetidase and Ldt enzymes, and which play a key role in its PG cross-linking. Hence, *M. marinum* on treatment with AMC-DIO or AMC-DMT combinations showed severe morphological changes, suggesting its potential role in the inhibition of PG cross-linking enzymes.

The WHO recommends AMC along with the MEM as an add-on therapeutic option for the treatment of drug resistant TB, since clavulanic acid is only available in combination with AMX^41^. However, the major drawback of MEM is its intravenous administration, thrice a day, and which further requires prolonged hospitalization of the patient. The present work has the potential significance in which the combination of drugs, AMC-DIO (or AMC-DMT) showed significant *in vitro* anti-mycobacterial activity, and the drugs can be administered orally for an effective TB therapy.

### Synergistic effects of AMC and DIO (or DMT) drug combinations in *M. marinum* infected *Drosophila melanogaster* fly model

**Figure 5:**
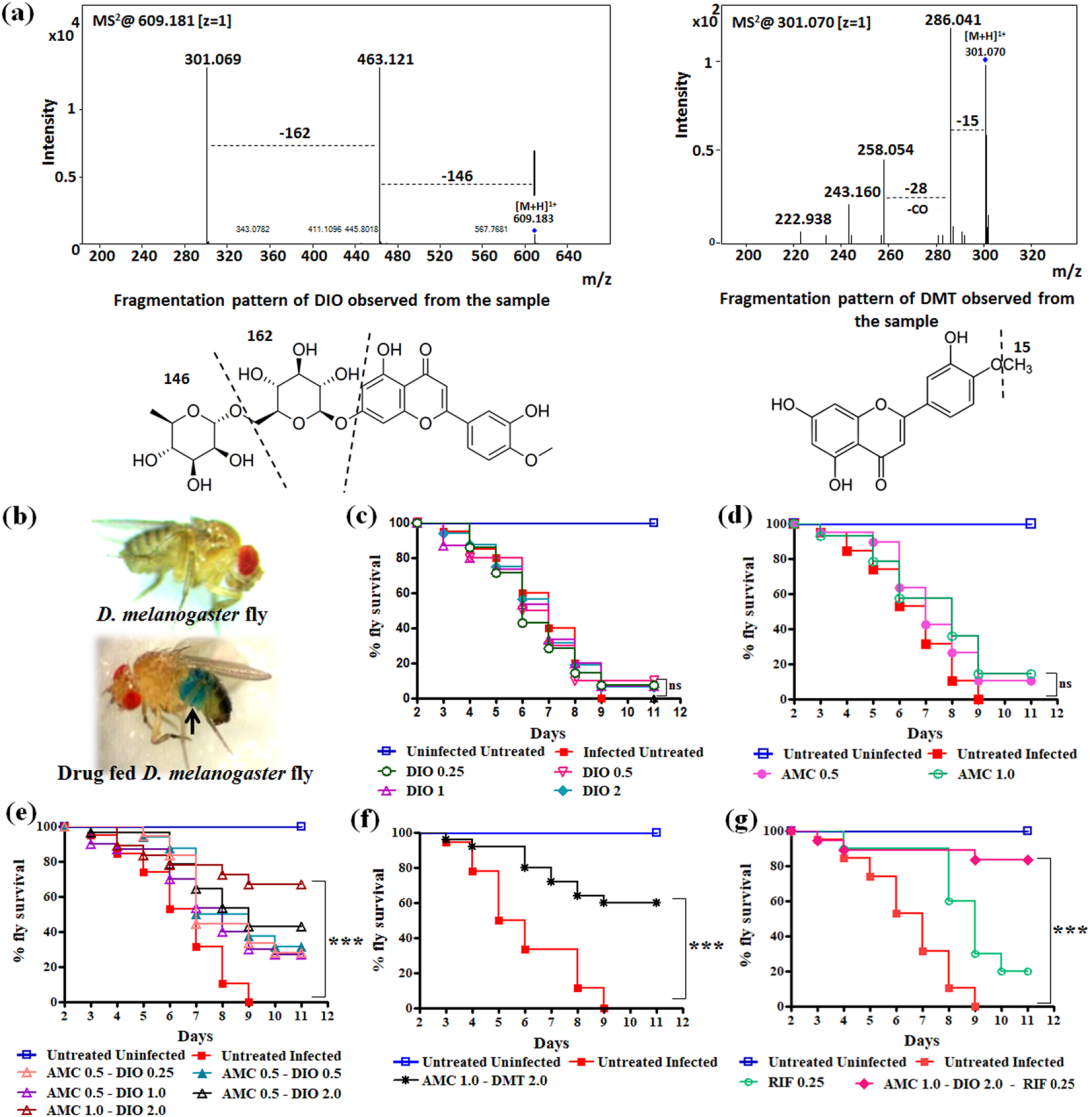
*In vivo* efficacy of AMC-DIO combinations in *M. marinum* infected *D. melanogaster* fly model. (**a**) The Q TOF LC-MS/MS spectrum of DIO fed fly extract indicating the conversion of DIO to DMT after oral administration. (**b**) The images of fly and fly fed with drug-food color (blue) mixture taken using stereo microscope (Magnüs MSZ-TR). Survival of *M. marinum* infected *D.melanogaster* flies on treatment with (**c**) DIO (0.25, 0.5, 1.0, and 2.0 mg/ml); (**d**) AMC (0.5 and 1.0 mg/ml) (**e**) AMC – DIO; (**f**) AMC-DMT; (**g**) Rifampicin (RIF; 0.25 mg/ml) and RIF along with 1 and 2 mg/ml of AMC and DIO respectively. The statistical significance was analysed using one way ANOVA (***p < 0.001; ns, not significant).

The *in vivo* efficacy of AMC-DIO and AMC-DMT combination of drugs were tested in *M. marinum* infected *D. melanogaster* fly model. Oh *et al*. reported *D. melanogaster*- *M. marinum* infection model for anti-mycobacterial activity assessment of different drugs^42^. Present *in vivo* studies of *M. marinum* infected flies showed 100% mortality within 9–10 days post-infection (Fig. 5b). It was reported earlier that in mammalian system, DIO gets converted into its aglycone form DMT by intestinal micro flora^34,35^. Thus, using mass spectrometry (MS) analysis, we confirmed that the conversion of DIO to DMT occurs in *D. melanogaster* flies after oral administration of DIO, as shown in Fig. 5a. Treatment of infected flies with different drug concentrations of either AMC or DIO individually does not show any marked fly survival (Fig. 5c,d). However, the survivability of the flies was significantly improved on treatment with increasing concentration of AMC and DIO combination. Maximum fly survival of 66.67% was observed at the higher concentration of AMC (1 mg/ml) and DIO (2 mg/ml) treated flies, revealing a synergistic effect of the combination of drugs (Fig. 5e).

Further, we analysed the efficacy of DMT (2 mg/ml) at the same concentration of DIO in combination with AMC (1 mg/ml) in the infected flies. AMC-DMT also exhibited a synergistic activity with a percentage survival of 60.0 of the infected flies, as shown in Fig. 5f. Thus, the present MS analysis and fly survival studies indicated that DIO after oral administration is converted into DMT. Also the therapeutic effect of either AMC-DIO or AMC-DMT is similar in *M. marinum* infected *D. melanogaster* fly model. Additionally, we checked the efficacy of AMC-DIO along with the standard TB drug, RIF. When infected flies were fed with a lower concentration of RIF (0.25 mg/ml), no significant fly survival was observed. Interestingly, a better fly survival of 83.33% was observed when infected flies were treated with a combination of AMC (1 mg/ml), DIO (2 mg/ml) and RIF (0.25 mg/ml), as shown in Fig. 5g.

**Figure 6:**
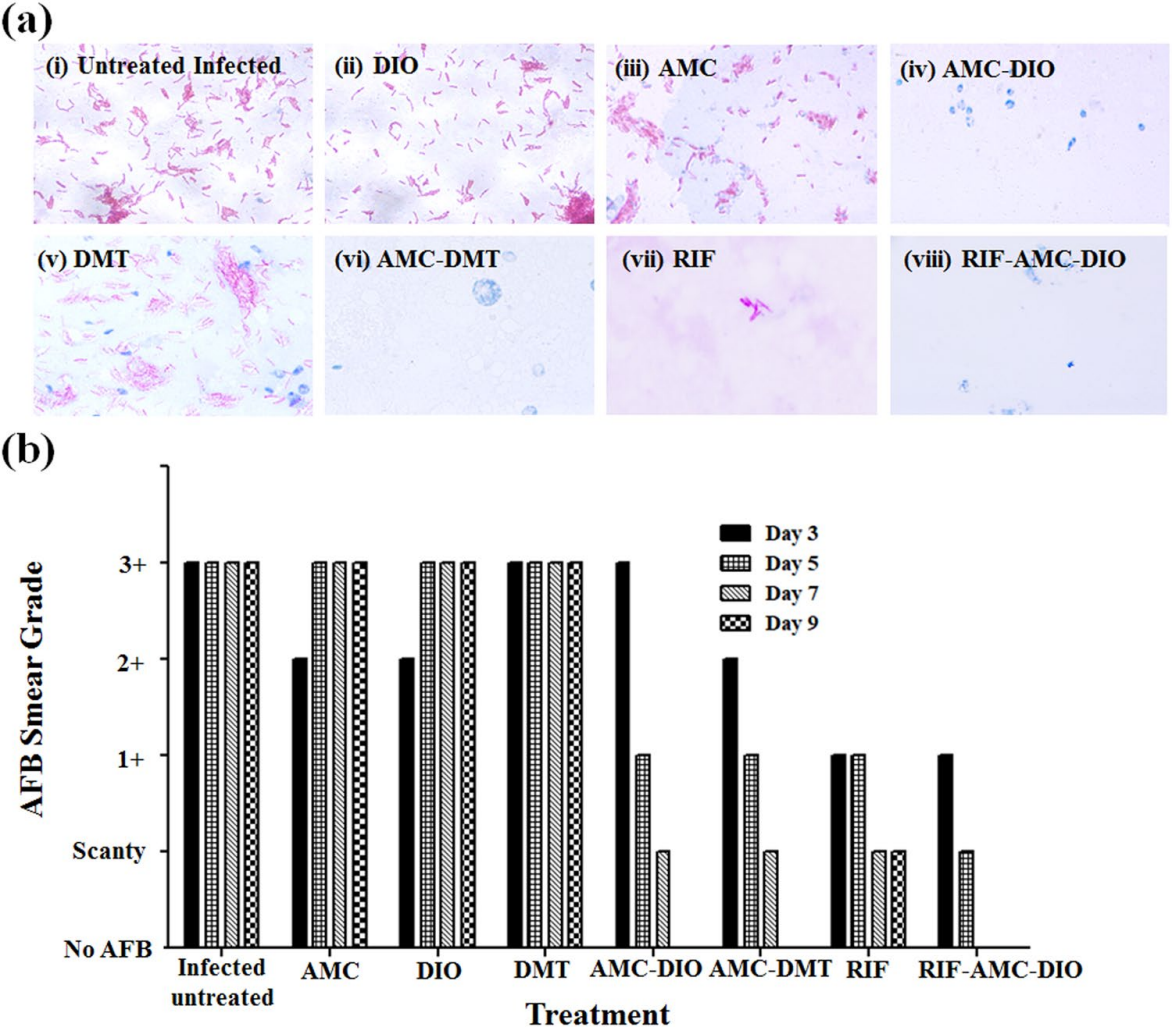
Bacterial load in the *M. marinum* infected *D. melanogaster* flies. (**a**) Acid fast bacilli (AFB) staining of *M. marinum* infected flies after 9 days of treatment (i) without any treatment and treatment with (ii) DIO, (iii) AMC, (iv) AMC-DIO, (v) DMT, (vi) AMC-DMT (vii) RIF (viii) RIF-AMC-DIO. (**b**) AFB grading of the *M. marinum* infected flies during the drug treatment (3^rd^, 5^th^, 7^th^ and 9^th^ days) showed that no bacilli were found in infected flies after 9 days of treatment with AMC-DIO, AMC-DMT and RIF-AMC-DIO.

Additionally, the bacterial load in the infected flies on 3^rd^, 5^th^, 7^th^ and 9^th^ day of drug treatment were determined by AFB (Acid Fast Bacilli) staining technique, as shown in Fig. 6. The number of bacteria present in 100 fields was counted and graded according to WHO/IUATLD Quantification scale (Supplementary Table S1). Progressive increase in the severity of *M. marinum* infection was seen in the flies treated with AMC, DIO or DMT individually, shown in Fig. 6a(ii,iii and v). The corresponding AFB smear grading of infected flies treated with these drugs was escalated to 3+ within five days of treatment, as shown in Fig. 6b. However, a reduction in the bacterial load was observed on treatment with AMC-DIO or AMC-DMT. The flies on the 9^th^ day of treatment with AMC-DIO or AMC-DMT were completely free from *M. marinum* infection as indicated by the absence of bacilli in the smears (Fig. 6a(iv and vi) and Fig. 6b). These *in vivo* observations strongly support our earlier fly survival studies and revealed a synergistic anti-mycobacterial activity of the combination of drugs. Further, we checked the bacterial load in the infected flies treated with RIF alone and in combination with AMC-DIO. In RIF treated fly group after 9 days of treatment, only a few bacilli were present in the smear samples (Fig. 6a(vii)). However, the survival of infected flies was only ~20% as shown in Fig. 5g. Further, RIF-AMC-DIO was very effective and no bacilli were seen in the fly smear from 7^th^ day onwards, as presented in Fig. 6a,b(viii). In summary, treatment of *M. marinum* infected flies with the individual drugs did not show any marked improvement in their survival, while AMC-DIO or AMC-DMT or AMC-DIO-RIF showed better survival of infected flies.

### *In vitro* anti-mycobacterial activity of AMC-DIO combination of drugs against *Mtb* and MDR clinical isolate

**Figure 7:**
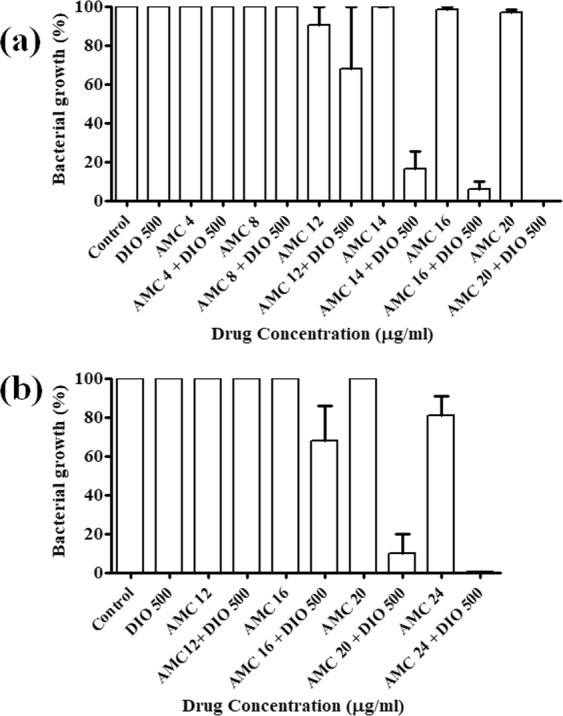
*In vitro* anti-mycobacterial activity of AMC, DIO and their combinations. Drug susceptibility testing of AMC-DIO combination against (**a**) *Mycobacterium tuberculosis* (*Mtb)* H37Ra and (**b**) Multi-drug resistant *Mtb* clinical isolate using MGIT 960 system, indicating a synergistic anti-tubercular effect of the drug combinations (AMC-DIO), whereas AMC or DIO alone showed no significant anti-mycobacterial activity.

Anti-mycobacterial activity of various concentrations of AMC, DIO and their combinations were tested against *Mtb* strain H37Ra using an automated MGIT 960 system. AMC (upto 20 μg/ml) and DIO (upto 500 μg/ml), when tested individually against *Mtb*, did not show any inhibitory activity. However, the combination of AMC (12 μg/ml) with 500 μg/ml of DIO was found to have a synergistic anti-mycobacterial effect. Increasing concentrations of AMC, 12 μg/ml to 20 μg/ml, when combined with 500 μg/ml of DIO, showed a gradual reduction in the mycobacterial growth (Fig. 7a). We observed a complete inhibition of mycobacterial growth at 20 μg/ml concentration of AMC in combination with 500 μg/ml of DIO, as shown in Fig. 7a.

Resistance to different antibiotics is of major concern in the current TB treatment regimen. Thus, we tested the *in vitro* efficacy of AMC and DIO both individually and in combination at different concentrations against MDR-*Mtb* clinical isolate. We observed that the individual drugs (AMC or DIO) were not able to inhibit the growth of MDR clinical isolate even at high concentrations (24 μg/ml of AMC; 500 μg/ml of DIO). However, AMC at concentrations above16 μg/ml, in combination with 500 μg/ml of DIO were able to show a concentration dependent reduction of mycobacterial growth. A complete growth inhibition of MDR clinical isolate was observed for AMC 24 μg/ml concentration in combination with 500 μg/ml of DIO, shown in Fig. 7b. Thus, the present *in vitro* efficacy studies clearly demonstrated a synergistic inhibitory activity of the AMC-DIO combination of drugs against a MDR clinical isolate of *Mtb*.

## Conclusions

Using computer-aided drug repurposing method we identified DIO by targeting both Ldt_Mt1_ and Ldt_Mt2_ enzymes of *Mtb*. After oral administration, DIO rapidly gets converted into its aglycone form, DMT. In the present study, we proved that both DIO and DMT interact with Ldt_Mt1_ and Ldt_Mt2_ enzymes using molecular docking technique and *in vitro* bioassay. A synergistic anti-mycobacterial activity of AMC and DIO or DMT combination was observed against *M. marinum* and *Mtb* H37Ra. Further, the *in vivo* efficacy of AMC-DIO or AMC-DMT combination of drugs was validated in *M. marinum* infected *D. melanogaster* fly model. Multi-drug resistance is a major challenge in the current TB combination therapy, leading to poor treatment outcomes. We demonstrated the synergistic anti-tubercular activity of AMC-DIO combination against a MDR-*Mtb* clinical isolate. The carbapenems are effective in killing both MDR and XDR strains of *Mtb*. However, they are not hydrolytically stable; this limits its antibiotic administration to a controlled intravenous infusion and also requires prolonged hospitalization. Both DIO and AMC are available as oral formulation, which can be a safe and inexpensive alternative for TB treatment. Further, DIO is a flavonoid compound with proven anti-oxidant, anti-inflammatory, chemo-preventive properties and it also modulates the host immune system. These pharmacological activities of DIO will thereby decrease the destruction of tissues and internal organs during *Mtb* infection. In conclusion, we have successfully repurposed DIO as an anti-mycobacterial agent in combination with AMC and which can be a potential alternative for the treatment of TB.

## Materials and Methods

### Computational Methods

Primary sequence analysis, 3D structural studies, molecular modelling, docking and MD simulation studies were performed on a Linux workstation using Schrödinger, AMBER and PyMol molecular modeling packages.

### Sequence and structure analyses of *Mtb* L,D-transpeptidases

The primary sequence of Ldt enzymes of *Mtb* (Ldt_Mt1_ and Ldt_Mt2_), *M. marinum* (Ldt_Mm1_ and Ldt_Mm2_) and *M. smegmatis* (Ldt_Ms1_ and Ldt_Ms2_), were retrieved from the UniProt sequence database and multiple sequence analysis (MSA) was performed using Clustal-X program^43^. Crystal structure of Ldt_Mt1_ (PDB ID: 4JMN)^19^ and Ldt_Mt2_ (PDB ID: 4GSR)^22^ were retrieved from Protein Data Bank (PDB) and the structures were optimized using the Protein Preparation Wizard module of Schrödinger^44^. Initially, the 3D structures of both Ldts were pre-processed by assigning the bond orders, adding hydrogen and deleting water molecules and finally the 3D structure minimization was done using OPLS2005 (Optimized Potential for Liquid Simulations) force field.

### Structure-based virtual screening using FDA approved drugs against Ldt_Mt1_ and Ldt_Mt2_

The chemical structures of all the FDA approved drugs (1556) was collected from the DrugBank database^45^ for our drug repurposing study. The geometry of these structures were optimized by employing MMFF94 (Merk Molecular Force Field) using the LigPrep wizard of Schrödinger^46^. Using GLIDE (Grid-based Ligand Docking with Energetics) module of Schrödinger v9.2^47^, molecular docking of these FDA approved drugs (1556) with the active sites of both *Mtb* Ldts was computed. Initially, centre of the grid box for molecular docking was selected by enclosing the experimentally reported active site residues for both Ldt_Mt1_ and Ldt_Mt2_ enzymes^19,22^. GLIDE docking program has been validated and successfully implemented in our previous VS as well as the host-pathogen interaction studies^48–50^. Molecular docking was performed in two stages: The first stage includes SP (standard-precision) docking followed by XP (extra-precision) docking with higher accuracy and less false positives. The GLIDE XP binding free energy is calculated using the following equation:

1 $${\rm{XP}}\,{\rm{Glide}}\,{\rm{score}}={{\rm{E}}}_{{\rm{coul}}}+{{\rm{E}}}_{{\rm{vdW}}}+{{\rm{E}}}_{{\rm{bind}}}+{{\rm{E}}}_{{\rm{penalty}}}$$

Where E_coul_, E_vdW_, and E_bind_ represent the electrostatic potential energy, van der Waals energy, and binding interaction energy respectively. E_penalty_ is the strain energy from protein, ligand or both, loss of entropy of the ligand and protein and desolvation of the ligand or protein^46^.

### Molecular dynamics simulation and binding free energy calculations of Ldt-drug complexes

Molecular dynamics (MD) simulations were performed using AMBER12^51^ program for 20 ns time interval for both Ldt_Mt1_, Ldt_Mt2_ proteins and three different protein-ligand complex systems- (i) Ldt_Mt2_-MEM, (ii) Ldt_Mt1_-DIO and (iii) Ldt_Mt2_-DIO. Initially, the atom types and the AM1-BCC charges for the ligands- MEM and DIO was assigned using Antechamber module and general amber force field (GAFF). The atom types of the proteins are assigned using the Leap program embedded in AMBER12, by employing the ff99SB force field. The uneven charge of these proteins was neutralized by adding sodium ions followed by solvation by immersing in a rectangular periodic box containing TIP3P water molecules. The prepared systems were then subjected to two step energy minimization employing steepest descent and conjugate gradient methods. These molecular systems were then subjected to initial equilibration, where it was heated from 0 to 300 K for 20 ps time interval with a cut-off of 10.0 Å for long range non-bonded interactions. The entire equilibration was performed using Langevin dynamics temperature control method and harmonic restraints was used with a force field constant of 10 kcal/mol Å^2^ to all the protein atoms. The MD production run without any restraints for 20 ns time interval was performed. Trajectory coordinates were recorded at every 250 ps of MD simulation and was analysed using PTRAJ module in AMBER12. Using MM-GBSA^52^ module, the binding free energy (ΔG_bind_) was calculated for Ldt_Mt2_-MEM (acting as a control for MD simulations), Ldt_Mt1_-DIO and Ldt_Mt2_-DIO complex systems.

## Experimental Methods

### Bacterial Strains and Growth Conditions

*Mtb* strain H37Ra (ATCC 25177) and *M. marinum* (ATCC 927), *E. coli* BL21 strains were used for this study. MDR clinical isolate of *Mtb* (AIMS 18/TC/130) was obtained from Department of Microbiology, AIMS, Kochi, India. This isolate was identified by BACTEC MGIT 960 as *Mtb*, resistant to all the first line anti-tubercular drugs (INH, RIF, Pyrazinamide, and Ethambutol) and second line quinolones (Ciprofloxacin, Ofloxacin, Levofloxacin). *M. marinum* and *Mtb* were grown in Middlebrook 7H9 broth supplemented with 0.05% Tween 80 and 10% albumin–dextrose complex (ADC) (Himedia) or in Middlebrook 7H11 solid medium supplemented with 10% ADC. *M. marinum* was cultured with 120 rpm shaking at 30 °C for 5 days. *Mtb* was cultured with 120 rpm shaking for 7–14 days at 37 °C. *E. coli* was grown in Luria-Bertani (LB) broth at 37 °C with 120 rpm shaking. All the experiments involving *Mtb* strain H37Ra and MDR clinical isolate were performed in a BSL-3 containment facility following institutionally approved protocols.

### Binding studies of Ldt_Mt1_ and Ldt_Mt2_ with DIO and DMT

The *E.coli* BL21 harbouring the plasmids pET2818 containing for *ldt*_*Mt1*_ gene (kindly gifted by Prof. Jean-Emmanuel Hugonnet, Pierre and Marie Curie University, Paris, France) and pET-21a(+) containing *ldt*_*Mt2*_ gene (kindly gifted by Prof. Se Won Suh, Seoul National University, Republic of Korea) were grown in LB broth to an optical density at 600 nm of ~0.5. The protein expression was induced by the addition of 1 mM Isopropyl β-D-1-thiogalactopyranoside and further incubated for 16 h at 22 °C. The cells were lysed by sonication in lysis buffer (50 mM NaH_2_PO_4_, 300 mM NaCl and 10 mM imidazole (pH 8.0)). The cell lysates were added into separate nickel-nitrilotriacetic acid (Ni-NTA) columns, pre-equilibrated with lysis buffer. The columns were washed with two column volumes of wash buffer containing 50 mM NaH_2_PO_4_, 300 mM NaCl and 20 mM imidazole (pH 8.0). DIO/DMT solution (50 µg/ml) was added into the Ni-NTA columns and subsequently washed with wash buffer. The proteins (Ldt_Mt1_and Ldt_Mt2_) were then eluted using the same buffer with 250 mM concentration of imidazole. The eluted protein fractions were incubated at 60 °C for 10 mins and the proteins and DIO (Mw: 609.181 Da) or DMT (301.071 Da) in the respective elutes were separated by ultrafiltration using Amicon Ultra-4 10 K centrifugal filter devices (Millipore). The optical density of the respective filtrates at 263.5 nm and 344 nm was determined for DIO and DMT respectively using Nanodrop Microvolume UV-Vis Spectrophotometer (Thermo Scientific). *E.coli* BL21 alone was used as the control for the experiment.

### *In vitro* anti-mycobacterial activity assays

(i) **Determination of MBC of AMC, DIO and DMT against** *M. marinum.* A stationary phase *M. marinum* culture was diluted to OD = 0.1 at 600 nm (corresponding to 0.3 × 10^7^ CFU/ml). The diluted culture was added into different test tubes containing Middlebrook 7H9 broth supplemented with 10% ADC in the ratio of 1:100 ^53^. Increasing concentrations of AMC (0.5, 1.0, 2, 4, 8 and 16 µg/ml; pre-dissolved in double distilled water at stock concentration of 10 mg/ml; where AMX and clavulanic acid are in the ratio 4:1), DIO (100, 250 and 500 µg/ml; predissolved in DMSO at stock concentration of 50 mg/ml) and their combinations were added in the test tubes and incubated for 3 days. Then the bacteria were plated onto Middlebrook 7H11 agar plates and the plates were incubated in an inverted position in a static incubator for colony enumeration. Similarly the MBC of AMC, DMT (500 µg/ml) and their combinations were determined against *M. marinum*. MBC was defined as the lowest concentration of the drugs that kills 99% of the mycobacteria.
(ii) **Mycobacterial susceptibility testing of AMC, DIO and DMT against** *Mtb* **using Mycobacterial Growth Indicator Tube (MGIT).** Anti-mycobacterial activity of AMC, DIO and its combinations were tested against *Mtb* strain H37Ra and a MDR clinical isolate using Becton Dickinson’s BACTEC MGIT 960 instrument^54^. H37Ra and MDR clinical isolate were inoculated into the MGIT tubes supplemented with antibiotics consisting of Polymyxin B, Amphotericin B, Nalidixicacid, Trimethoprim, and Azlocillin (MGIT PANTA Antibiotic mixture from Becton Dickinson) and oleic acid-albumin–dextrose complex (OADC) enrichment broth (Becton Dickinson). Different concentrations of AMC (2, 4, 8, 12, 14, 16, 20 and 24 µg/ml; where AMX and clavulanic acid is in the ratio 4:1) and DIO (500 µg/ml) drugs were added individually and in combinations to these MGIT tubes, and incubated at 37 °C. Mycobacterial cultures without any drugs were used as its growth control. The fluorescence intensity of all the MGIT tubes were recorded when the growth controls were flagged positive.

### Scanning electron microscopy study

Two days old *M. marinum* cultures were treated with AMC (8 µg/ml; AMX and clavulanic acid in the ratio 4:1), DIO (500 µg/ml), DMT (500 µg/ml) and their combinations for 48 h. Samples were fixed using 2.5% (v/v) glutaraldehyde and dehydrated in graded ethanol series^55,56^. Samples were gold sputter coated and imaged to study mycobacterial morphology.

### Mass Spectrometry study

*Drosophila melanogaster* flies were raised in standard banana agar medium and allowed to feed DIO (2 mg/ml) for 5 days. Flies were then homogenized with a pestle in 100 μl of sterile double deionized water and extracts were used for MS studies. The Q-TOF LC-MS/MS analysis were carried out using an Agilent 1290 Infinity Ultra High Performance Liquid Chromatographic system coupled to an Agilent 6540 UHD Accurate Mass Q-TOF mass spectrometer equipped with Dual AJS (ESI) source. The samples were infused to the mass spectrometer through a reversed-phase column (Agilent Poroshell-120, 2.1 × 150 mm, 2.7 µm). The mobile phase (A: water with 0.1% formic acid; B: acetonitrile with 0.1% formic acid) flow rate was maintained at 0.2 ml/min. A linear gradient of 5–95% mobile phase B in 20 min was used to separate the molecules. The mass spectra were acquired between 100 and 1000 m/z and the dry gas flow and dry gas temperature were set at 6 ml/min and 320 °C, respectively. Targeted MS/MS data of DIO (theoretical monoisotopic protonated mass, 609.181 Da), and DMT (theoretical monoisotopic protonated mass, 301.071 Da) were collected by setting the collision energies at 20 V and 25 V, respectively. All the MS data were acquired in positive ionization mode using Agilent Mass Hunter data acquisition software, Vers. B.05.01 and the results were analyzed using Agilent Mass Hunter qualitative analysis software, Vers. B.07.01^57^. Solvents (acetonitrile, water and formic acid) used for MS study was of HPLC grade and procured from Merck.

### *In vivo* anti-mycobacterial activity studies in *M. marinum* infected *D. melanogaster* fly model

*In vivo* experiments were carried out using 5 to 7 days old male *D. melanogaster* fly (Flybase Stock 2:FBst0000002). Flies were fed and raised at 26 °C on banana agar media with 60% humidity^58^. One day prior to the infection, the flies were starved for 6 h. A Whatman filter paper disc of 2.3 cm diameter was placed on top of the banana medium in glass vials and 60 µl of cyclophosphomide (stock concentration: 500 µg/ml, dissolved in double distilled water) was added to the filter paper disc. Flies were then transferred to these glass vials and allowed to feed. After 24 h, these flies were injected with *M. marinum* (OD_600_ = 3.0) at the junction area between the ventral and dorsal cuticles of the fly, using needle of 31 Gauge thickness. Flies were then returned to the standard fly culture vials containing respective drugs, AMC (AMX and clavulanic acid in the ratio 4:1), DIO, DMT, and RIF; the combination of drugs, AMC-DIO, AMC-DMT and AMC-DIO-RIF respectively. A Whatman filter paper disc was placed on the banana agar surface of the fly culture vials and 60 µl of different concentrations of specific drugs, AMC (0.25, 0.5 and 1 mg/ml), DIO (0.25, 0.50, 1 and 2 mg/ml), DMT (2 mg/ml) and RIF (0.25 mg/ml) and their combinations were added to the filter paper disc. The flies were transferred to fresh media and fly survival was recorded for 11 days post infection. Each of these fly experiments were repeated thrice using 30 flies/group (n = 30) and the Kaplan-Meier survival plots were generated.

Further, the infected flies treated with drugs as mentioned above were collected on 3^rd^, 5^th^, 7^th^and 9^th^ days post infection to determine the bacterial load. Fly smears were prepared and then stained with Ziehl-Neelsen method for acid fast bacilli, visualized and graded under the microscope in accordance with the WHO/IUATLD Quantification scale^59^ (Supplementary Table S2).

### Statistical Analysis

All the experiments were performed in triplicate and the results were represented as an average ± standard deviation. Statistical analyses were carried out using GraphPad Prism5 software. p < 0.05 (*)p < 0.01 (**) and p < 0.001 (***) were considered statistically significant.

## Supplementary information

Combination of Repurposed Drug Diosmin with Amoxicillin-Clavulanic acid Causes Synergistic Inhibition of Mycobacterial Growth
