# Supplementary material for: Combination of Repurposed Drug Diosmin with Amoxicillin-Clavulanic acid Causes Synergistic Inhibition of Mycobacterial Growth
Source: Sci Rep. 2019 May 1;9:6800. doi: 10.1038/s41598-019-43201-x (PMC6494880; doi:10.1038/s41598-019-43201-x)
Supplement: Supplementary file 1 — Combination of Repurposed Drug Diosmin with Amoxicillin-Clavulanic acid Causes Synergistic Inhibition of Mycobacterial Growth [file 41598_2019_43201_MOESM1_ESM.doc]

**Supplementary Information**

**Combination of Repurposed Drug Diosmin with Amoxicillin-Clavulanic acid Causes Synergistic Inhibition of Mycobacterial Growth**

### Anju Choorakottayil Pushkaran^†^, Vivek Vinod^†^, Muralidharan Vanuopadath^+^, [**Sudarslal Sadasivan Nair**^+^**,**](https://www.amrita.edu/faculty/sudarslal) Shantikumar V Nair^†^, Anil Kumar Vasudevan ^#^, Raja Biswas^†*^, Chethampadi Gopi Mohan^†*^

Center for Nanosciences and Molecular Medicine^†^,

Department of Microbiology^#^

Amrita Institute of Medical Sciences and Research Centre,

Ponekkara, Kochi-682 041,

School of Biotechnology^+^,

Clappana, Kollam- 690 525,

Amrita Vishwa Vidyapeetham,

Kerala, India

Corresponding Authors*

Dr. C Gopi Mohan and

Dr. Raja Biswas

Center for Nanosciences and Molecular Medicine

Amrita Vishwa Vidyapeetham

E-mail: [cgmohan@aims.amrita.edu](mailto:cgmohan@aims.amrita.edu)

[rajabiswas@aims.amrita.edu](../rajabiswas@aims.amrita.edu)


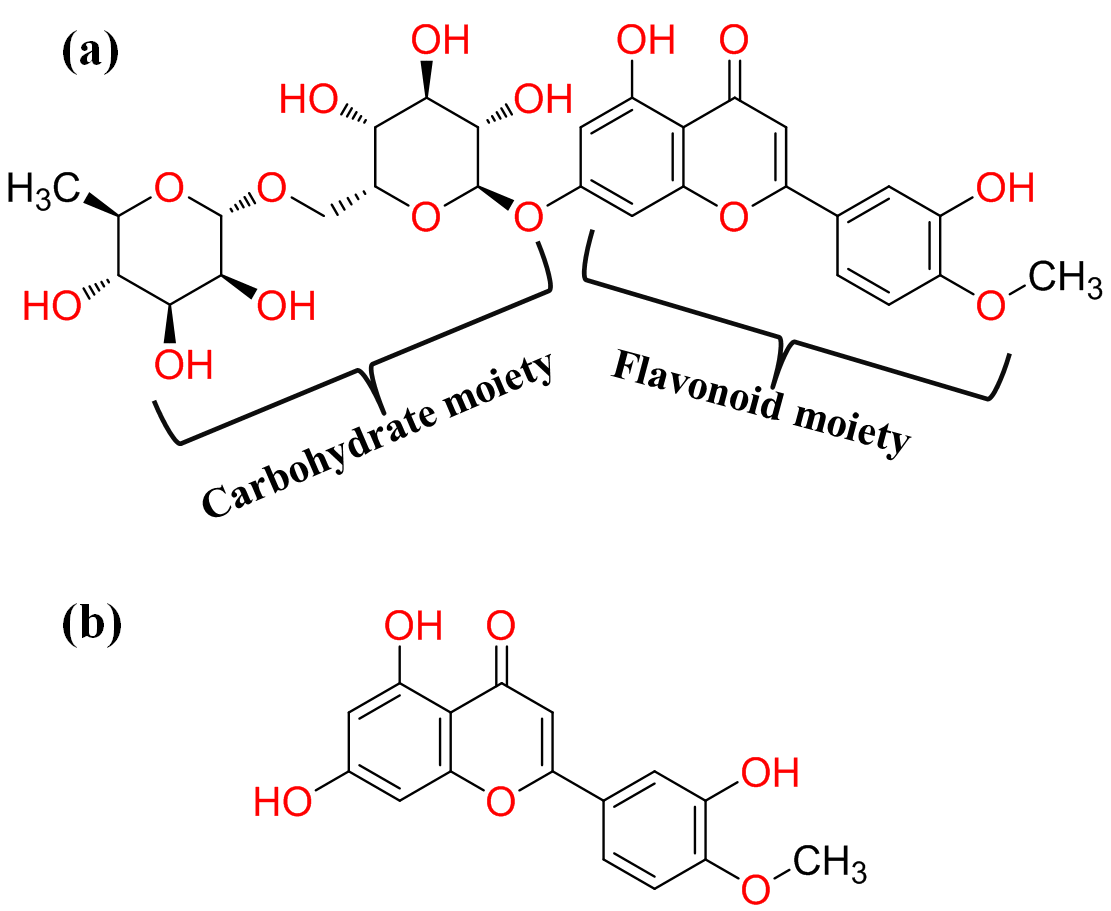


**Figure S1**. Structure of (a) Diosmin (DIO) and its metabolite (b) Diosmetin (DMT).


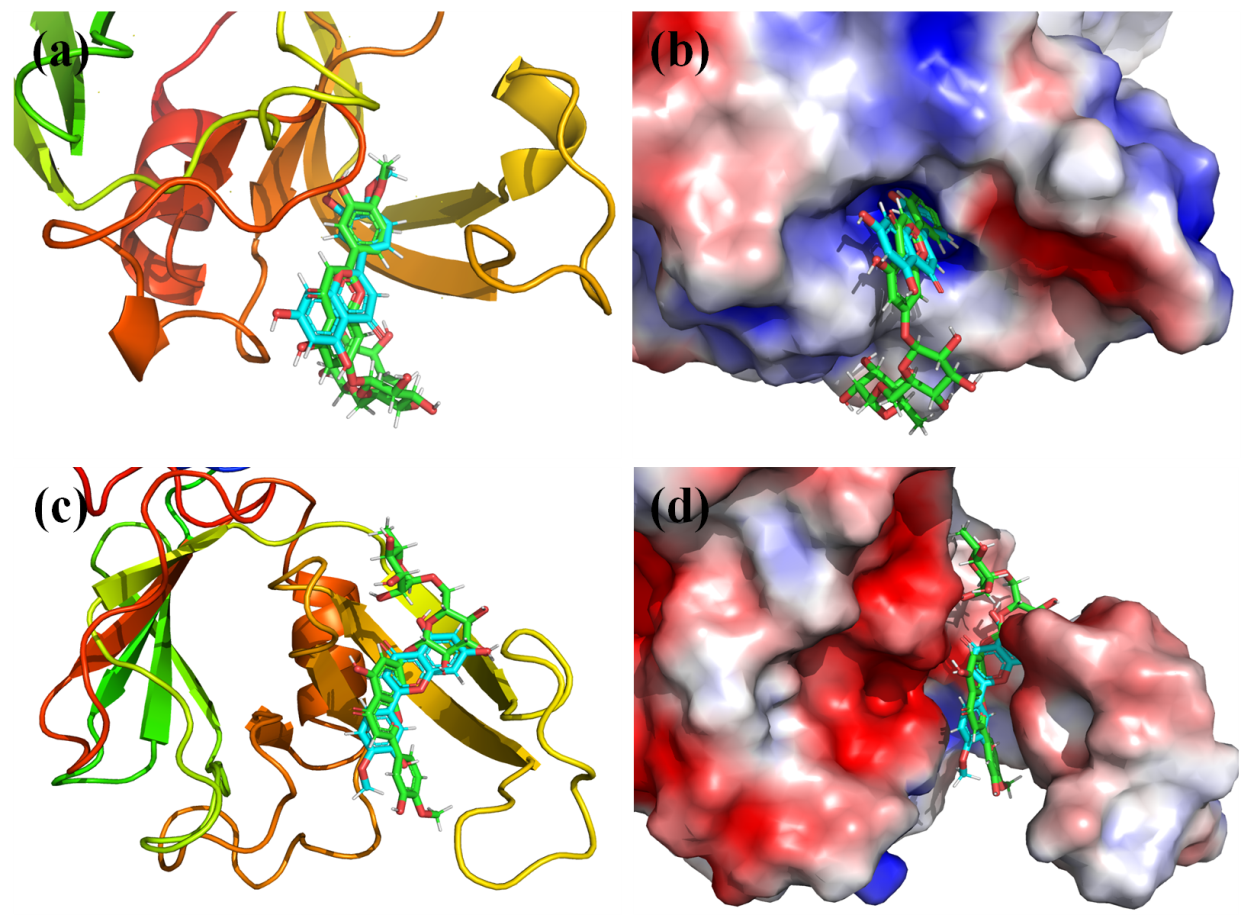


**Figure S2**. Superposition of docked complexes of Ldt_Mt1_ and Ldt_Mt2_ with DIO and DMT. (a) Superposition of Ldt_Mt1_ -DMT complex with the binding pose of DIO; (b) The MEP surface map of the active site region of Ldt_Mt1_ with DMT (Cyan sticks) and DIO (green sticks) binding pose; (c) Superposition of Ldt_Mt2_ -DMT complex with the binding pose of DIO; (d) The MEP surface map of the active site region of Ldt_Mt2_ with DMT (Cyan sticks) and DIO (green sticks) binding pose.


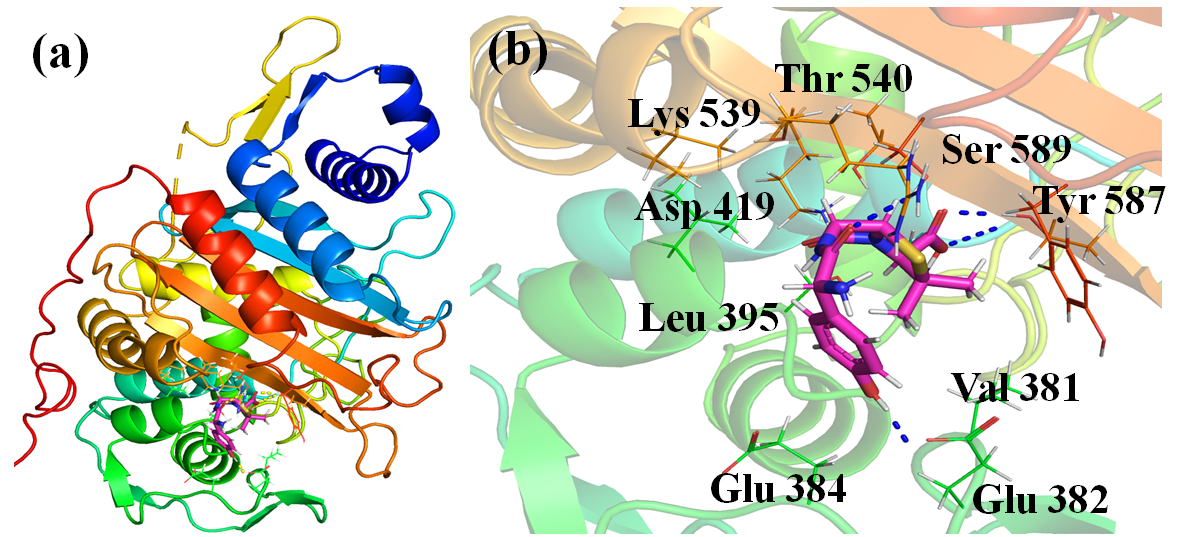


**Figure S3:** (a) Docked complex of D,D-transpeptidase enzyme in complex with Amoxicillin (AMX) and (b) enlarged view of its active site showing molecular interactions to the drug. The hydrogen bonding interaction is shown as blue dotted lines and the amino acid residues at the active sites are represented as lines.

**Table S1**: MM/GBSA binding free energy calculations for Ldt-DIO and Ldt-Diosmetin complexes.

|  | | **Diosmin** | **Diosmetin** |
| --- | --- | --- | --- |
| **Glide score**  **(kcal/mol)** | Ldt_Mt1_ | -9.37 | -6.48 |
|  | Ldt_Mt2_ | -10.55 | -6.29 |
| **∆G_bind_**  **(MM/GBSA)**  **(kcal/mol)** | Ldt_Mt1_ | -30.61 | -25.83 |
|  | Ldt_Mt2_ | -30.01 | -19.73 |
| **∆E_vdW_**  **(kcal/mol)** | Ldt_Mt1_ | -46.76 | -34.68 |
|  | Ldt_Mt2_ | -53.04 | -29.40 |
| **∆E_ele_**  **(kcal/mol)** | Ldt_Mt1_ | -9.83 | -15.25 |
|  | Ldt_Mt2_ | -19.39 | -5.57 |
| **∆G_gas_**  **(kcal/mol)** | Ldt_Mt1_ | -56.59 | -49.92 |
|  | Ldt_Mt2_ | -72.44 | -34.98 |
| **∆G_solv_**  **(kcal/mol)** | Ldt_Mt1_ | 25.98 | 24.09 |
|  | Ldt_Mt2_ | 42.42 | 15.25 |

ΔG_bind_ = ΔH - TΔS ≈ ΔE_MM_ + ΔG_solv_ – TΔS

ΔE_MM_ = ΔE_internal_+ ΔE_electrostatic_ + ΔE_vdW_

ΔG_solv_= ΔG_GB_ + ΔG_SA_

where ΔE_MM_, ΔG_solv_ and -TΔS denotes the changes of the gas phase MM energy, the solvation free energy, and the conformational entropy upon binding, respectively. ΔE_MM_ includes ΔE_internal_ (bond, angle, and dihedral energies), ΔE_electrostatic_ (electrostatic), and ΔE_vdw_ (van der Waals) energies. ΔG_solv_ is the sum of electrostatic solvation energy (polar contribution), ΔG_GB_, and the non-electrostatic solvation component (non-polar contribution), ΔG_SA_. The conformational entropy change -TΔS is computed by the normal-mode analysis on a set of conformational snapshots taken from the MD simulation trajectories.

**Table S2.** Grading of the smears for Ziehl-Neelsen staining (WHO/IUATLD Quantification scale[^1^](#_ENREF_1))

| **Number of Acid Fast Bacilli (AFB)** | **Number of fields examined*** | **Grading** |
| --- | --- | --- |
| No AFB | 100 | No AFB |
| 1-9 AFB | 100 | Scanty |
| 10-99 AFB | 100 | 1+ |
| 1-10 AFB in each field | 50 | 2+ |
| >10 AFB in each field | 20 | 3+ |

*Oil immersion fields

**References**

1. Laszlo A. Sputum examination for tuberculosis by direct microscopy in low income countries*. IUATLD Technical Guide International Union against Tuberculosis and Lung Disease, Paris, Franc*e 2000.

**************************************
